# Spontaneous Regression and Separation of Idiopathic Epiretinal Membranes

**DOI:** 10.7759/cureus.44473

**Published:** 2023-08-31

**Authors:** Ruchir Gupta, Heather Leslie, Yi Zhang

**Affiliations:** 1 Ophthalmology, Lewis Katz School of Medicine at Temple University, Philadelphia, USA; 2 Ophthalmology, Temple University Hospital, Philadelphia, USA

**Keywords:** vitreomacular traction, optical coherence tomography, regression, separation, posterior vitreous detachment (pvd), epiretinal membrane

## Abstract

Epiretinal membranes (ERMs) typically remain stable or progressively worsen. Here, we report two rare cases of spontaneous ERM regression and one case of ERM separation. This is a case series of three patients. The patients’ clinical data were collected and ERMs were evaluated with serial optical coherence tomographies (OCTs).

Cases one and two were female patients presenting with floaters. Mild-to-moderate idiopathic ERM was diagnosed which spontaneously regressed over the following years without intervention and evidence of separation. Patients’ vision was slightly decreased or remained stable, respectively. Case three was a female patient presenting with blurry vision. A mild ERM was diagnosed with vitreomacular traction (VMT) and a tiny macular hole. Over the following three months, ERM separation along with VMT release was evidenced on OCT. The macular hole healed simultaneously. Her vision improved from 20/70 to 20/30.

The possibility of spontaneous regression of idiopathic ERMs should be a factor to consider in management. Further research must be done to determine the mechanism of this phenomenon.

## Introduction

An epiretinal membrane (ERM) is composed of two layers overlying the internal limiting membrane. The outermost layer consists of non-cellular extracellular matrix proteins, and the innermost layer contains myofibroblastic pre-retinal cells that reach the retinal surface via defects in the internal limiting membrane or from the vitreous cavity [1]. The prevalence of ERM is 7-11.8%, with the major risk factor being increased age. ERMs can be divided into idiopathic and secondary causes. The most common association with idiopathic ERM formation is posterior vitreous detachment (PVD). The most common cause of secondary ERM formation is prior cataract surgery [2].

Treatment generally consists of vitrectomy with membrane peeling if symptomatic. In rare instances, spontaneous separation of ERM has been observed [3-13]. Even more rarely, the ERM has been noted to spontaneously regress without signs of separation [14-17]. Here, we present two cases of idiopathic ERM that regressed spontaneously without separation and one case of spontaneous idiopathic ERM separation.

## Case presentation

Case one: spontaneous regression of idiopathic epiretinal membrane

A 66-year-old female with a past medical history of migraines, hyperlipidemia, coronary artery disease, cervical cancer, chicken pox, measles, mumps, and a two-year history of PVD in both eyes (OU) presented with floaters OU for one week and pain in the right eye. She reported blurry vision in the mornings, without flashes. There was no history of ophthalmic surgeries. Visual acuity (VA) was 20/20 in the right eye (OD), and 20/20 in the left eye (OS). Intraocular pressure (IOP) was within normal limits. Slit lamp examination was unremarkable except for 1+ nuclear sclerosis OU. Fundus examination was indicative of PVD OU, mild ERM OD over macula, and blunted macular reflex OS. No optical coherence tomography (OCT) was performed at this visit.

**Figure 1:**
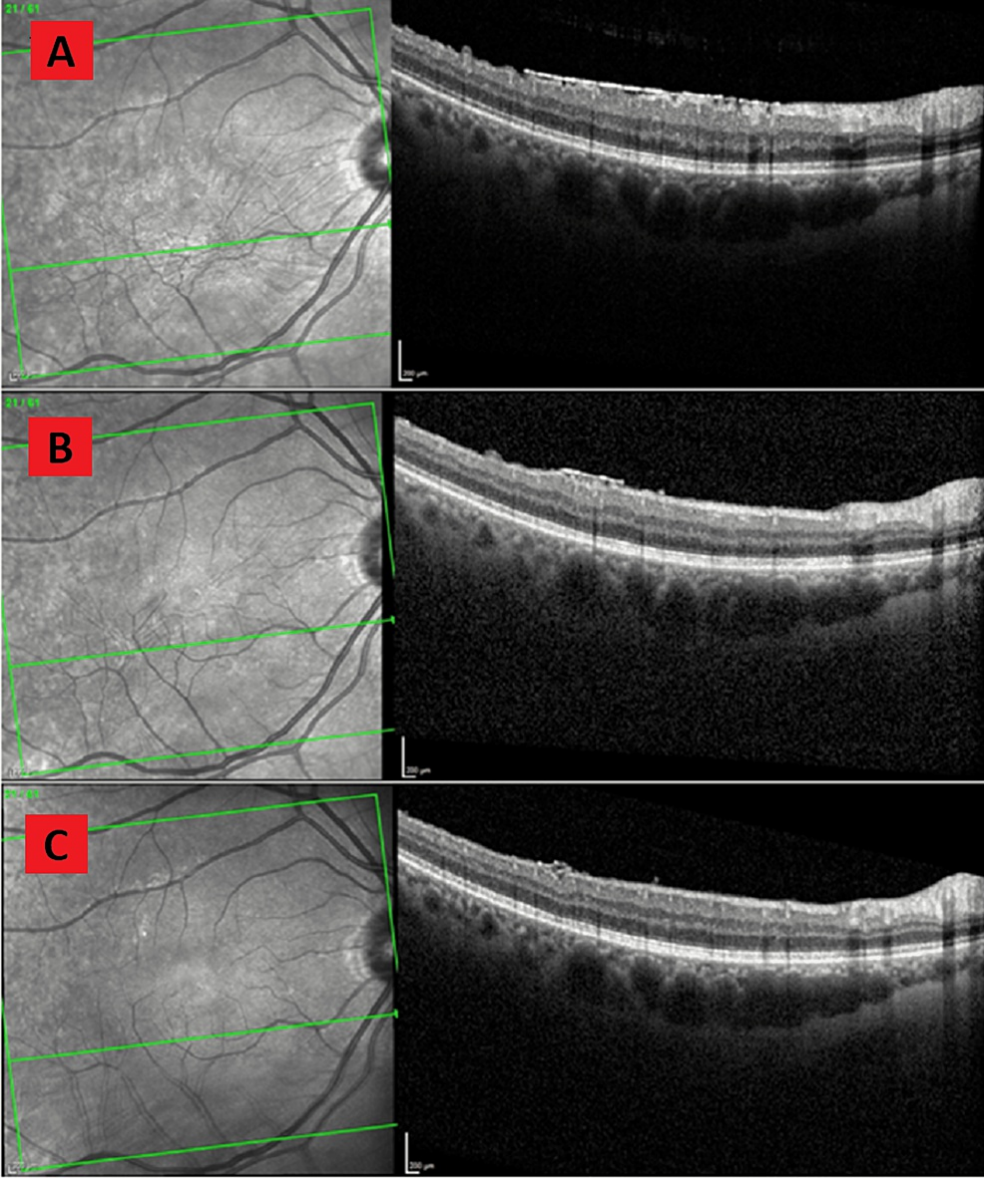
Serial OCT evaluations showing spontaneous regression of idiopathic ERM in case one. 1A: OCT six months after ERM diagnosis showing moderate ERM over the fovea causing the irregular surface of the retina. 1B: OCT 13 months after diagnosis showing significantly regressed ERM and resolution of retinal surface irregularities. 1C: OCT 22 months after diagnosis showing only trace ERM, retina restored to almost regular structure, and smooth surface. OCT: optical coherence tomography; ERM: epiretinal membrane

At the sixth-month follow-up visit, the patient reported stable floaters. VA was 20/20 OD and 20/25 OS. Fundus examination was indicative of lattice degeneration OU. OCT was indicative of moderate ERM OD without edema (Figure 1A). At the 13th-month follow-up, the patient reported stable floaters. VA was 20/25 OD and 20/20 OS with pinhole. The slit lamp and fundus examinations were unchanged from before. OCT was indicative of regressing ERM compared to the previous OCT (Figure 1B). At the 22nd-month follow-up, the patient reported stable floaters. VA was 20/30 OD and 20/25 OS with pinhole. The slit lamp and fundus examination were unchanged from the previous findings. OCT was indicative of further ERM regression (Figure 1C).

Case two: spontaneous regression of idiopathic epiretinal membrane

**Figure 2:**
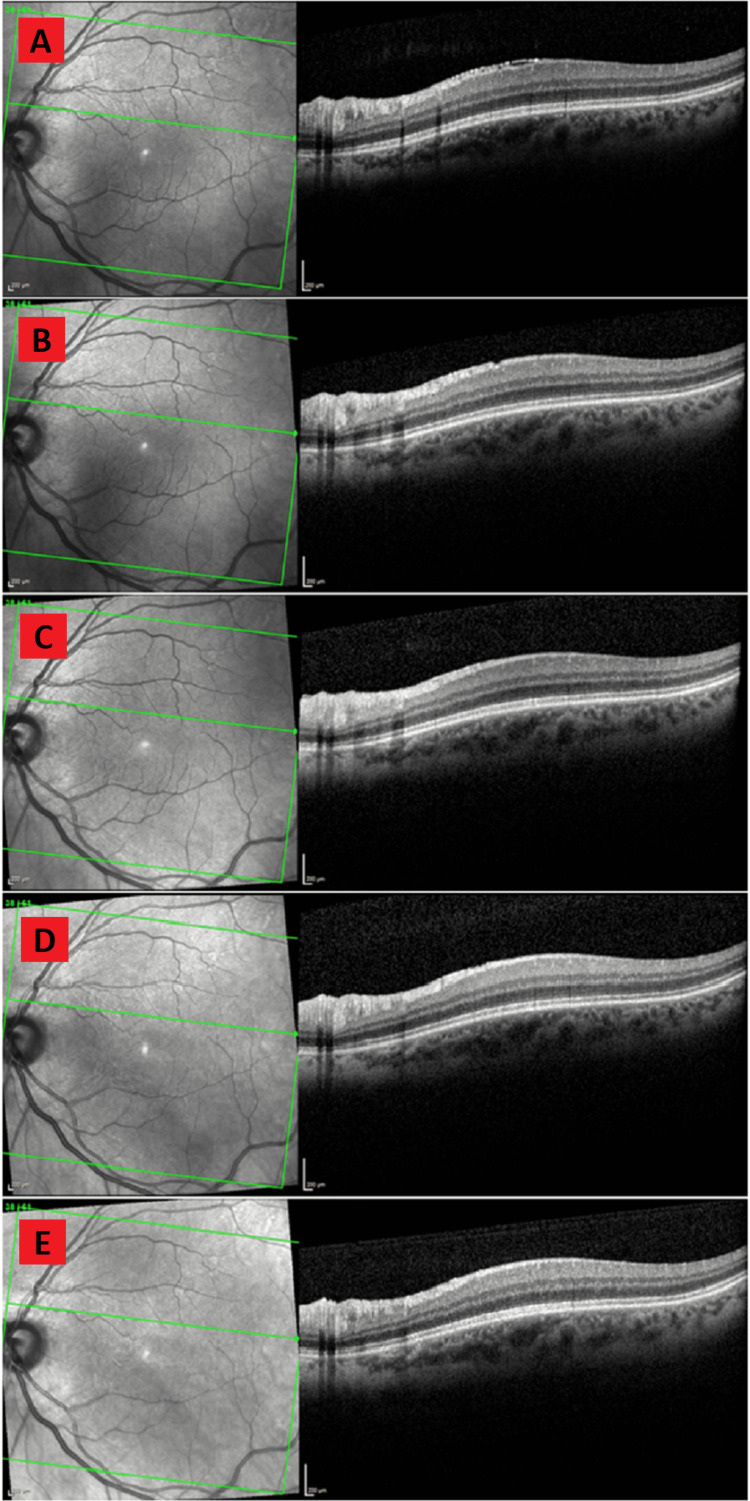
Serial OCT evaluations showing spontaneous regression of idiopathic ERM in case two. 2A: OCT at the time of the initial ERM diagnosis showing mild ERM in the macula. 2B: OCT 15 months after the diagnosis showing slight ERM regression. 2C: OCT 27 months after the diagnosis showing further ERM regression. 2D: OCT 40 months after the diagnosis showing trace remains of regressing ERM. 2E: OCT 51 months after the diagnosis showing complete ERM resolution. OCT: optical coherence tomography; ERM: epiretinal membrane

A 62-year-old female with no known past medical history presented with constant floaters OS for the past two years. The patient had no complaints of flashes, pain, and blurred vision. There was no history of ophthalmic surgeries. VA with pinholes was 20/25 OD and 20/25 OS. IOP was within normal limits. The slit lamp examination was indicative of trace nuclear sclerosis OU. Fundus examination was indicative of PVD OS, and mild ERM OS. OCT was indicative of mild ERM OS (Figure 2A). At the 15th-month follow-up, the patient reported floaters OU for the past nine months. The patient had mild blurry vision and no flashes. VA with pinholes was 20/40 OD and 20/40 OS. The slit lamp examination was indicative of additional PVD OD. Fundus examination and OCT were indicative of mild ERM OS which was stable/improved compared to the previous OCT (Figure 2B). At the 27th-month follow-up, the patient denied floaters, flashes, pain, and blurred vision. VA with pinholes was 20/30 OD and 20/30 OS. The slit lamp and fundus examinations were unchanged from the previous examination. Trace ERM OS was present on OCT which was further improved compared to the previous findings (Figure 2C). At the 40th-month follow-up, the patient had floaters OU but denied flashes or blurry vision. VA was 20/30 OD and 20/25 OS. The slit lamp and fundus examination were unchanged from the previous examination. OCT was indicative of trace ERM OS (Figure 2D). On follow-up at 51 months, VA was 20/25 OD and 20/25 OS. The slit lamp examination was indicative of 1+ nuclear sclerosis but was otherwise unchanged from the previous examination. The fundus examination was unchanged from the previous examination. OCT was indicative of complete ERM regression OS (Figure 2E).

Case three: spontaneous separation of idiopathic epiretinal membrane

**Figure 3:**
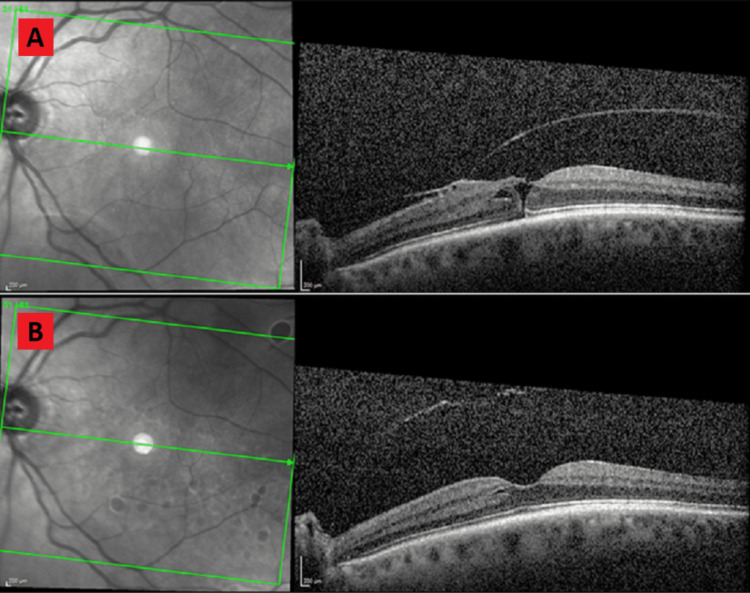
Serial OCT evaluations showing separation of idiopathic ERM in case three. 3A: OCT at the time of the initial diagnosis showing mild ERM overlying macula with VMT and a tiny macular hole. 3B: OCT three months after the diagnosis showing VMT release, self-healed macular hole, and resolved ERM. OCT: optical coherence tomography; ERM: epiretinal membrane; VMT: vitreomacular traction

A 66-year-old female with a past medical history of osteoporosis, iron deficiency anemia, gastroesophageal reflux disease, and rheumatoid arthritis on hydroxychloroquine presented with blurry vision OS. She had no complaints of eye pain, floaters, and flashes. She has had no prior ophthalmic surgeries. VA with pinholes was 20/30 OD and 20/70 OS. IOP was within normal limits. The slit lamp examination was indicative of 1+ nuclear sclerosis. Fundus examination was indicative of mild ERM overlying macula OS. OCT was indicative of vitreomacular traction (VMT), mild ERM, and tiny macular hole OS (Figure 3A). At the third-month follow-up, the patient denied blurriness OS. VA with pinholes was 20/40 OD and 20/30 OS. The slit lamp examination was unchanged. The fundus examination was indicative of resolved ERM. OCT was indicative of complete VMT release, self-healed macular hole, and resolved ERM (Figure 3B).

## Discussion

Idiopathic ERMs typically remain stable or progressively worsen, and it is quite unusual for resolution to occur naturally without intervention. Spontaneous idiopathic ERM separation, as described in case 3, is rare clinically but has been reported in the literature several times [3-13]. The following three mechanisms for spontaneous ERM separation have been proposed [11]: (A) ERM is pulled away by a PVD; (B) contracting forces of the ERM cause separation from edges toward the center; (C) acute detachment of ERM at its weakest central point and retraction of part of the membrane toward the epicenter. Case three appears to be an example of mechanism A as OCT images suggest that the ERM separated from the retina along with the posterior hyaloid membrane likely due to the VMT.

Spontaneous idiopathic ERM regression, as described in cases one and two, is even more unusual with only a few reports. The regression observed in cases one and two appears to be a dissolution rather than a peeling as there is no OCT evidence of the ERM separating as a whole, rather the ERM appears to thin and disappear gradually. Typically, this type of regression may occur with the resolution of inflammation in ERMs that is secondary to an acute inflammatory process such as trauma, infection, or uveitis [18]. Our cases involved idiopathic ERMs and had no history of an underlying inflammatory process making the resolution particularly unusual.

The current literature on spontaneous regression of ERM without signs of separation includes five cases [14-17]. Three of them involve ERMs secondary to an inflammatory process, and two of them involve idiopathic ERMs. One of these cases was associated with optic nerve atrophy [16]. Another case involved bilateral spontaneous ERM regression [17]. The case of spontaneous regression presented by Schadlu et al. involved a pre-existing PVD [15]. Sheybani et al. demonstrated that idiopathic ERMs contain retinal pigment epithelium cells, whereas inflammation-associated ERMs do not, indicating that the two membranes are distinct entities [19]. Evidence of spontaneous regression in both inflammatory ERMs and idiopathic ERMs indicates that there may be multiple mechanisms for spontaneous regression. Further research must be done to determine the mechanism behind spontaneous regression of idiopathic ERM.

## Conclusions

We have presented one case of separation of idiopathic ERM and two cases of regression of idiopathic ERM. The case of separation had OCT evidence of separation and was thought to be due to PVD and VMT release. The mechanism behind the cases of regression is unknown as this is a rarely reported event. ERMs secondary to an inflammatory process may regress as the underlying inflammatory process resolves, but this mechanism could not occur in the cases we present here as these ERMs are idiopathic. Elucidation of the mechanism of ERM regression may help identify the cause of the disease and prompt future therapies to induce such an event. The possibility of ERM regression occurring spontaneously, especially in patients with pre-existing PVDs, should be a factor to consider in management.
